# Time to Resumption of Menses, Spatial Distribution, and Predictors Among Post-partum Period Women in Ethiopia, Evidence From Ethiopian Demographic and Health Survey 2016 Data: Gompertz Inverse Gaussian Shared Frailty Model

**DOI:** 10.3389/frph.2022.862693

**Published:** 2022-05-13

**Authors:** Daniel Gashaneh Belay, Melaku Hunie Asratie

**Affiliations:** ^1^Department of Human Anatomy, College of Medicine and Health Sciences, University of Gondar, Gondar, Ethiopia; ^2^Department of Epidemiology and Biostatistics, College of Medicine and Health Sciences, Institute of Public Health, University of Gondar, Gondar, Ethiopia; ^3^Department of Women's and Family Health, School of Midwifery, College of Medicine and Health Sciences, University of Gondar, Gondar, Ethiopia

**Keywords:** Ethiopia, Gompertz inverse Gaussian shared frailty, menses, post-partum, resumption

## Abstract

**Background:**

The timing of the resumption of post-partum menses is important for a woman who intends to avoid subsequent unintended pregnancy, and it has key implications on maternal, neonatal, and child health outcomes. Despite this, information is scant about the time to resumption of post-partum menses and predictors in Ethiopia. Therefore, this study aimed to determine the time it takes to start menses and spatial distribution among post-partum period women in Ethiopia and identify its predictors.

**Methods:**

A secondary data analysis was conducted based on 2016 Ethiopian Demographic and Health Survey (EDHS). A total weighted sample of 6,489 post-partum women was included in the analysis. STATA 14 was used to weigh, clean, and analyze the data. The shared frailty model was applied since the EDHS data have a hierarchical nature. For checking the proportional hazard assumption, the Schenefold residual test, Log-Log plot, Kaplan–Meier, and predicted survival plot were applied. Akakie Information Criteria (AIC), Cox–Snell residual test, and deviance were used for checking model adequacy and for model comparison. Based on these, the Gompertz inverse Gaussian shared frailty model was the best-fitted model for this data. Variables with a *p* < 0.2 were considered for the multivariable Gompertz inverse Gaussian shared frailty model. Finally, the adjusted hazard ratio (*AHR*) with a 95% confidence interval (*CI*), and a *p* < 0.05 was reported to identify the significant predictors of time to the resumption of post-partum menses.

**Results:**

The median survival time to post-partum menses resumption was 14.6 months. In this study, 51.90% [95% *CI*: 50.03, 53.76] of post-partum period women had resumed, and the risk of menses resumption was 1.17 times [*AHR*: 1.17; 95% *CI*: 1.03–1.33] higher among urban resident, 1.14 times [*AHR*: 1.14; 95% *CI*: 1.0–1.24] in women who had attended formal education, and 1.63 times [*AHR*: 1.63; 95% *CI*: 1.4–1.7] higher among women who used hormonal contraceptives. However, the risk of post-partum menses resumption was lower among 7–24 months breastfeeding women by 36% [*AHR*: 0.64; 95% *CI*: 0.5–0.76], women with child alive by 26% [*AHR*: 0.74; 95% *CI*: 0.6–0.85], and multiparous women by 27% [*AHR*: 0.73; 95% *CI*: 0.6–0.80].

**Conclusion:**

Almost half of the participants had resumed post-partum menses, with the median survival timing of menses resumption at 14.5 months. Women residing in urban areas, who attended formal education, and using hormonal contraceptives have a shorter time to resume post-partum menses, whereas a woman with an alive child, breastfeeding practice, and multiple parity has a longer time to resume post-partum menses. Therefore, the healthcare providers and program managers should act on the resumption of post-partum menses through health education and promotion to cultivate the 14 months lag period identified by considering the significant factors.

## Background

The post-partum period is full of time in terms of both physiological and anatomical changes in women (1, 2). Such women should expect some bleeding and vaginal discharge after giving birth, and they must characterize it to differentiate whether it is lochia or menses. In the first week of the post-partum period, women might experience heavier and clot bleeding, then followed by bodily fluid that can appear clear to creamy white to red in color called lochia (3, 4). When the discharge had the appearance of lochia, stopped for some time, and followed by bright red bleeding, this is the resumption of post-partum menses (5). A post-partum period woman returning to the menstrual cycle is just one of the parts of recovery and returning to their pre-pregnancy body. The time of resumption of post-partum menses is highly dependent on the hormonal conditions of the women (6, 7). As reported from a finding, the length of time between the resumption of post-partum menses is used as a proxy determinant of fertility (8). Post-partum menses is different from the pre-pregnancy period like that of women's life changed after having a baby (9). This is the hidden challenge in Ethiopia with limited evidence. Currently, there is a research question that needs to be covered timely by this study “What is the median time of resumption of post-partum menses, and the determinant factors among post-partum period women in Ethiopia?”

Having concrete data about the cut point of time of resumption post-partum menses is especially important to avert a substantial number of maternal, neonatal, and child mortality from different perspectives (10). Both direct and indirect complications of unintended pregnancy, adverse consequences of the short interbirth interval, and puerperal infection are the three current challenges for developing countries to reduce maternal, neonatal, and child mortality (11–13). Different studies showed that giving attention to the time of post-partum menses resumption is the weapon to tackle the three challenges (14, 15).

There is evidence that shows a lack of adequate knowledge on the time of post-partum menses resumption, and the use of post-partum contraceptives is not directly associated with unintended pregnancy (16). Shreds of evidence show that unintended pregnancy secondary to not used post-partum contraceptive is the tip of an iceberg in the developing country with a lack of adequate information about the timing of resumption of post-partum menses (17, 18). Recently published data about the prevalence of unintended pregnancy in Ethiopia showed 27.9% (19), 23.5% (20), 41.5% (21), and 26.6% (22), and convincing evidence from systematic review shows the overall prevalence is 28% (23). Once an unintended pregnancy occurs, it endangers the women from complications of unsafe abortion (24). On the other hand, unintended pregnancy, secondary to lack of adequate knowledge on the time of resumption of post-partum menses, and not using contraceptives among post-partum period women, takes the lion's share of maternal, neonatal, and child death due to the consequences of a short interbirth interval (25, 26). In the context of Ethiopia, short interbirth intervals contribute to 30% of maternal death by predisposing them to bleeding disorders during pregnancy, post-partum hemorrhage, complications of anemia, and developing psychological problems (27, 28). All those above-listed challenges due to the complications of unintended pregnancy and adverse consequences of the short interbirth interval can be alleviated by directly acting on the time of resumption of post-partum menses in coordination with the scale-up of post-partum family planning services (29).

From clinical experience, most of the women with unintended pregnancies reported that the reason for the pregnancy was that they had not used post-partum contraceptives as their post-partum menses had not resumed. They believe that conception will not occur until their post-partum menses are resumed. Most women are not familiar with the occurrence of pregnancy in the absence of post-partum menses resumption.

Using the Locational Amenorrhea Method (LAM) as the family planning procedure (30, 31) plays an important role in clearly showing the cut point of time of resumption of post-partum menses. Locational amenorrhea is the preferred family planning method for those post-partum period women with medical complications and curious follow-up on the ovulation period/time of menses resumption (32). To implement locational amenorrhea as a family planning method, the women should be on exclusive breastfeeding, their menses should not be started, and they must be within 6 months of the postpartum period (33, 34). Unintended pregnancy is inevitable unless the post-partum period women are knowledgeable about these three criteria. Therefore, there is a need to have plenty of evidence about the time of the resumption of post-partum menses as it is important for the intervention.

As reported from previous studies, there are different factors affecting the timing of post-partum menses resumption, such as complete breastfeeding (35), breastfeeding more frequently, on-demand, for a longer period including at night (36), the timing of the introduction of dietary supplements (36), women with HIV infection have an unexplained prolonged resumption of menses more often than at-risk seronegative women (37), and bottle feeding is associated with an early resumption of post-partum menstruation and ovulation (38).

Though the resumption of post-partum menstruation is an important indicator of fertility after birth, information is scant about the commonly agreed cut point of time for the resumption of post-partum menses. Therefore, the current study was aimed to determine the median time to resumption of post-partum menses, spatial distributions, and identified predictors among post-partum period women in Ethiopia, based on 2016 EDHS data. Having plenty of evidence about the median time of the resumption of post-partum menses, and its predictors are very crucial to healthcare providers for the recommendation of ovulation period during post-partum counseling, and program managers might take it as a baseline data to design strategies for the best outcome of women's, neonatal, and child health. Furthermore, understanding the significant hotspot areas of post-partum menses resumption would help to evaluate the diverse cultural practices of breastfeeding and other proximal determinants of post-partum menses resumption.

## Methodology

### Study Design, Area, and Data Source

Population-based cross-sectional survey data from EDHS 2016 were used for this study. Ethiopia (3o−14o N and 33o - 48E) is an east African country with 1.1 million km^2^ coverage and an estimated population of 100,613,986, which makes it the second-most populous country in Africa. Administratively, Ethiopia is federally decentralized into nine regions (Afar, Amhara, Benishangul-Gumuz, Gambela, Harari, Oromia, Somali, Southern Nations, Nationalities, and People's Region (SNNPR), and Tigray) and two cities administrative (Addis Ababa and Dire-Dawa). Regions are divided into zones, then into districts and kebeles. Kebele is the lowest administrative unit and is subdivided into census enumeration areas (EAs), which are convenient for the implementation of the census. The EDHS used a stratified two-stage cluster sampling technique selected in two stages using the 2007 Population and Housing Census (PHC) as a sampling frame. Stratification was achieved by separating each region into urban and rural areas. In total, 21 sampling strata have been created. In the first stage, 645 enumeration areas (EAs) (202 in the urban area) were selected with a probability of selection proportional to the EA (enumeration areas) size and independent selection in each sampling stratum. In the second stage, on average, 28 households have systematically selected. The details of the study design and setting are available elsewhere (36).

### Study Population

All women who had children <36 months (about 3 years) and who had records for the time to resumption of postpartum menses during the survey (EDHS 2016) were included in the study population. The total weighted sample of 6,489 women was included in the analysis.

### Study Variables

The outcome variables of the study were the time to resumption of postpartum menses which was measured in months. A woman is considered as an event (having postpartum menses) if the woman has menses after delivering the last child during the study period, else is considered censored. The independent variables considered for this study were socio-demographic and obstetrical related variables, such as the age of mother, sex of the child, duration of breastfeeding, child alive status, parity, family planning usage, maternal education, occupation of mother, wealth status, and media exposure of the household and residence.

### Operational Definitions

**Event (postpartum menses resumptions):** a woman who **resumes** menses after the delivery of her last child during the study period (39). The amenorrhea status is from the mother's report of her status at the time of the interview. For all other births, the mother is assumed not to have amenorrhea since the birth (40).

**Time:** the time interval in months from the delivery of the last child up to the date of conception of the following child or the date of interview if there was no following birth.

**Censored:** the women who have not had menses resumed after the delivery of their last child during the study period.

### Data Processing and Analysis

The data were accessed in STATA format after being registered as an authorized user. STATA 14 was used for data clearance and analysis. The data were weighted using sampling weight before any statistical analysis to restore the representativeness of the survey. The data clearance, descriptive, and summary statistics were conducted using STATA version 14 software. The random effect of the survival model was checked to assess the clustering effect since the EDHS data have a hierarchical structure where households are nested within-cluster/EAs, which violates the assumption of the independence of observations and equal variance across clusters. The theta parameter was used to assess whether there is significant clustering or not. The Schoenfeld residual test and Log-Log plot were applied to check the proportional hazard (PH) assumptions. The log-likelihood ratio test, deviance (-2LL), and Akakia Information and Criteria (AIC) were used to select a model. A model with the highest values of log-likelihood and the lowest value of AIC was the best-fitted model. Deviance and AIC showed that the Gompertz inverse Gaussian shared frailty model had the lowest value which was the best-fitted model for the data.

Variables with a *p* < 0.20 in the uni-variable Gompertz inverse Gaussian shared frailty analysis were included in the multivariable analysis. In the multivariable analysis, the adjusted hazard ratio (*AHR*) with a 95% confidence interval (*CI*) was used to declare significant predictors for the time to resumption of menses.

## Results

### Socio-Demographic Characteristics of the Study Population

A total weighted 6,489 women were included in this study; nearly half of the study participants found in the age group 25–34 years were 3,350 (51.64%). Most of the study participants 5,738 (88.43%) were living in rural areas and more than half of the women 5,738 (62.23) had no formal education (Table 1).

**Table 1:** Socio-demographic characteristics of the study population in Ethiopia, 2016 Ethiopian Demographic and Health Survey (EDHS).

**Variables**	**Categories**	**Weighted**	**Percentage**
		**frequency**	**(%)**
Age of women	15–24	1,774	27.34
	25–34	3,350	51.64
	35–49	1,364	21.02
Sex of child	Male	3,266	50.34
	Female	3,222	49.66
Breast feeding duration	Never	279	4.30
	Ever	1,761	27.15
	0–6 months	1,321	20.36
	7–24 months	2,524	38.90
	25–35 months	603	9.29
Child alive	No	309	4.76
	Yes	6,180	95.24
Women education status	No education	4,038	62.23
	Formal education	2,450	37.77
Occupation of mother	Not working	3,668	56.53
	Working	2,820	43.47
Wealth index	Poor	3,031	46.71
	Middle	1,318	20.31
	Rich	2,139	32.98
Media exposure	No	4,293	66.15
	Yes	2,196	33.85
Parity	Primiparous	1,162	17.91
	Multiparous	5,326	82.09
Family planning	Not used	4,491	69.22
	Hormonal	1,850	28.52
	Non-hormonal	146	2.26
Residence	Urban	751	11.57
	Rural	5,738	88.43
Region	Tigray	454	7.00
	Afar	67	1.03
	Amhara	1,183	18.24
	Oromia	2,877	44.35
	Somalia	299	4.60
	B/gumiz	69	1.06
	SNNPR	1,322	20.38
	Gambella	15	0.23
	Harare	16	0.24
	Addis ababa	159	2.45
	Dire dawa	27	0.42

### Obstetrical Related Characteristics of Participants in Ethiopia, 2016 EDHS

Among 6,489 women 3,266 (50.34%) of them were with the sex of a male child, and 2,524 (38.9%) of them cultivated the culture of breastfeeding practice for 7–24 months (about 2 years). Women with children alive in this study accounted for 6,180 (95.24%), 5,326 (82.9%) of them were multipara, and 4,491 (69.22%) of them were not using hormonal contraceptives (Table 2).

**Table 2:** Obstetrical related characteristics of participants in Ethiopia, 2016 EDHS.

**Variables**	**Categories**	**Weighted frequency**	**Percentage (%)**
Sex of child	Male	3,266	50.34
	Female	3,222	49.66
Breast feeding duration	Never	279	4.30
	Ever	1,761	27.15
	0–6 months	1,321	20.36
	7–24 months	2,524	38.90
	25–35 months	603	9.29
Child alive	No	309	4.76
	Yes	6,180	95.24
Parity	Primiparous	1,162	17.91
	Multiparous	5,326	82.09
Family planning	Not used	4,491	69.22
	Hormonal	1,850	28.52
	Non-hormonal	146	2.26

### Resumption of Postpartum Menses of the Respondents

**Figure 1:**
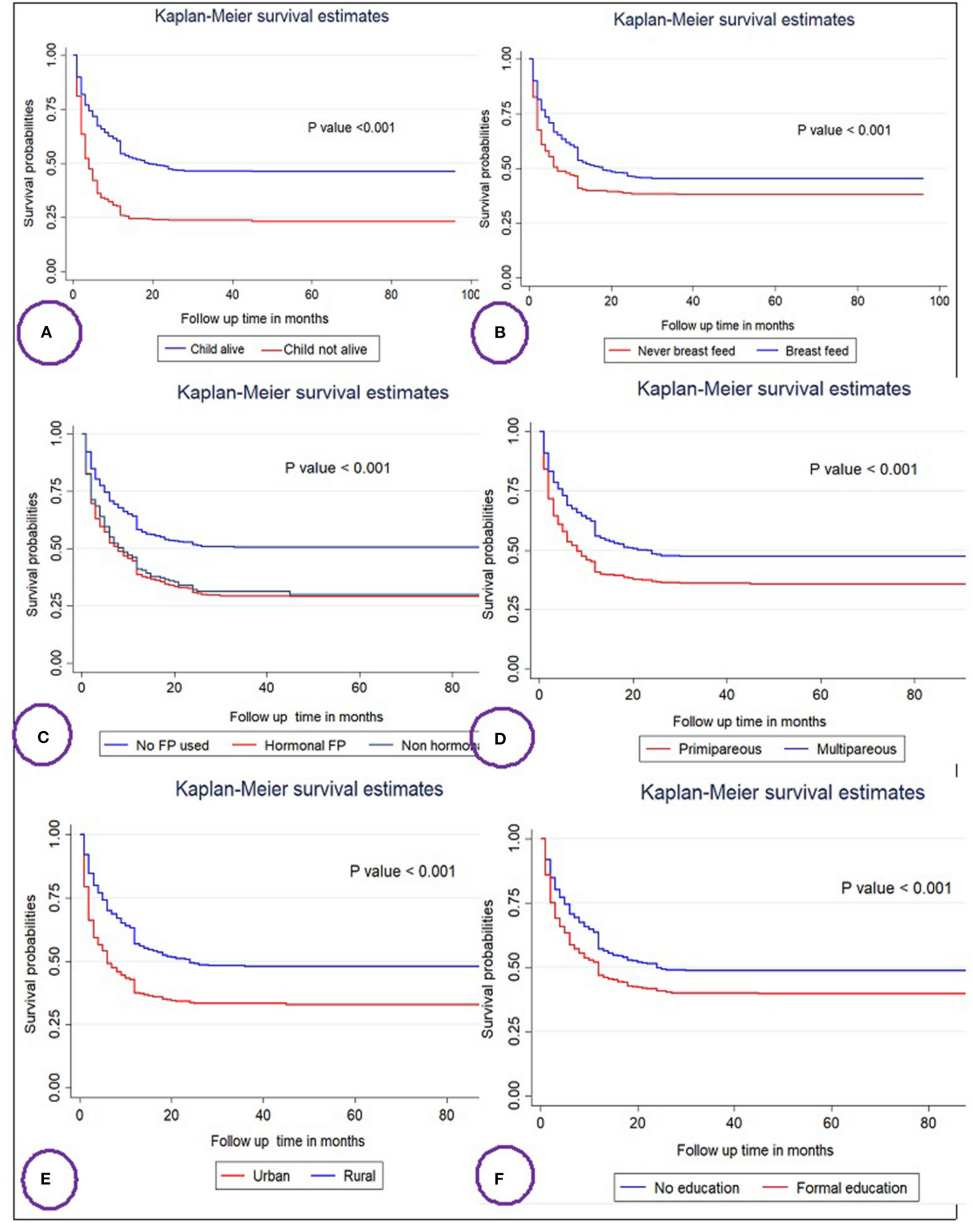
Kaplan–Meier survival curves and log rank tests of the time to resumption of postpartum menses among women's in Ethiopia based on child status **(A)**, breast feeding **(B)**, family planning (FP) usage **(C)**, parity **(D)**, residence **(E)**, and educational status **(F)**.

Of the total 6,489 studied women, 51.90% [95% *CI*: 50.03, 53.76] had postpartum menses resumption, the rest 48.09% [95% *CI*: 46.23, 49.96] of them had no post-partum menses (they are censored) during the follow-up time. The total follow-up time contributed by all study participants was 19,189 person-years. The overall median survival time was 14.6 months. The median survival time varies by the characteristics of respondents. The median survival time, for example, in urban areas was 5.7 months, whereas in rural areas it was 15.3 months (Figure 1).

### Predictors of Time to Resumption of Postpartum Menses

Comparisons of survival functions of different categorical variables.

Differences in key variables at baseline among various categories were determined using the log-rank test and the Kaplan–Meier survival function. The Kaplan–Meier survival function was constructed for different categorical variables. In general, the pattern of the survivorship function lying above another indicated that the group defined by the upper curve (blue color) had a longer survival (short-time failure) than the group defined by the lower curve (red color). For instance, mothers who breastfeed have longer survival than their counterparts who breastfeed mothers at log-rank *p* < 0.001 (Figure 1B). The significance of the graphically observed difference was assessed by a log-rank test and is indicated in the *p*-value of respected figures (Figure 1).

### Model Selection and Checking PH Assumptions

The proportional hazard (PH) assumption was assessed by Shenfield residuals. The Shenfield residuals test result showed that *p* < 0.001 with a Chi-square value of 82.06, which is significant. This smallest value of *p* is evidence to contradict the PH assumption. Therefore, a parametric type of model must be fitted. Based on deviance, AIC, and the Cox–Snell residual test, the shared frailty model with Gompertz distribution and inverse Gaussian frailty was the most efficient model, since it had the lowest deviance and AIC value (Table 3).

**Table 3:** Model diagnostics and comparison for the time to resumption of postpartum menses and predictors among reproductive age women in Ethiopia.

**Models**	**Distribution**	**Frailty**	**Theta**	**AIC**	**BIC**	**Deviance**
						**(−2LL)**
Shared frailty	Gompertz	Gamma	0.038	17010	17212	16950
Shared frailty	Gompertz	Inverse Gaussian	0.039	17009	17211	16948
Shared frailty	Exponential	Gamma	0.515	23206	23401	23148
Shared frailty	Exponential	Inverse Gaussian	0.801	23191	23386	23132
Shared frailty	Weibull	Gamma	0.118	20247	20449	20188
Shared frailty	Weibull	Inverse Gaussian	0.126	20248	203450	20188
Shared frailty	Log-Normal	Gamma	0.093	19521	19723	19460

In the Gompertz inverse, Gaussian shared frailty model variables with *p* < 0.2 in the bi-variable analysis were considered for multivariable analysis. Based on this, variables, such as duration of breastfeeding, child alive status, maternal education, parity, hormonal contraceptive usage, and residence, were significant predictors of the resumption of post-partum menses in the multivariable analysis.

The risk of resumption of post-partum menses was 36% lower in women who have breastfed for more than 7–24 months (about 2 years) than in women who have not breastfed [*AHR* = 0.64, 95% *CI*: 0.5, 0.76]. Women with a live child have a 26% lower risk to resume their menses during postpartum than that of women without a live child [*AHR* = 0.74, 95% *CI*: 0.6, 0.85].

The risks of having postpartum menses are 1.14 times higher among women with formal education than women with no education [*AHR* = 1.14, 95% *CI*: 1.0, 1.24]. Multiparous women had a 27% lower hazard of postpartum menses than primiparous women [*AHR* = 0.73, 95% *CI*: 0.6, 0.80]. Women who used hormonal contraceptives were at a 1.63 times higher risk of having postpartum menses compared with non-contraceptive users [*AHR* = 1.63, 95% *CI*: 1.4, 1.7]. Women who are living in urban areas have a 1.17 times higher risk of having early resumption of postpartum menses than women living in rural areas [*AHR* = 1.17, 95% *CI*: 1.03, 1.33] (Table 4).

**Table 4:** Shared frailty survival regression analysis of the time to resumption of postpartum menses and its predictors among women in Ethiopia.

**Variables**	**Categories**	**Menses resumption**	**CHR [95% CI]**	**AHR [95% CI]**	
		**Event *n* = 3,368 (51.90)**	**Censored *n* = 3,121(48.10)**		
Age of women	15–24	941 (53.04)	833 (46.96)	1.00	1.00
	25–34	1,747 (52.14)	1,604 (47.86)	0.87 [0.80, 0.94]**	0.98 [0.90, 1.08]
	35–49	680 (49.84)	684 (50.16)	0.78 [0.708, 0.868]***	0.94 [0.884, 1.055]
Sex of child	Male	1,807 (55.34)	1,458 (44.66)	1.00	1.00
	Female	1,560 (48.41)	1,662 (51.59)	0.91 [0.856, 0.981]*	0.94 [0.884,0.1.01]
Breast feeding duration	Never	155 (55.82)	123 (44.18)	1.00	**1.00**
	Ever	1,338 (75.98)	423 (24.02)	1.34 [1.142, 1.585]***	1.3 [1.17, 1.64]***
	0–6 months	220 (16.64)	1,102 (82.36)	0.19 [0.157, 0.236]***	0.2 [0.17, 0.26]***
	7–24 months	1,182 (46.83)	1,342 (53.12)	0.60 [0.509, 0.712]***	0.64 [0.5, 0.76]***
	25–35 months	472 (78.25)	131 (21.75)	**1.12 [0.923, 1.349]**	1.16 [0.92, 1.453]
Child alive	No	219 (71.12)	89 (28.88)	1.00	1.00
	Yes	3,148 (50.94)	3,032 (49.06)	0.47 [0.406, 0.537]***	0.74 [0.6, 0.85]***
Women education status	No education	1,957 (48.47)	2,081 (51.53)	1.00	1.00
	Formal education	1,411 (57.56)	1,040 (42.44)	1.35 [1.256, 1.455]***	1.14 [1.0, 1.24]**
Occupation of mother	Not working	1,808 (49.30)	1,860 (50.70)	1.00	1.00
	Working	1,559 (55.29)	1,261 (44.7)	1.09 [1.009, 1.171]*	1.04 [0.965, 1.124]
Wealth index	Poor	1,487 (49.08)	1,543 (50.92)	1.00	1.00
	Middle	636 (48.27)	682 (51.73)	1.01 [0.899, 1.122]	0.99 [0.891, 1.119]
	Rich	1,243 (50.13)	896 (41.87)	1.36 [1.253, 1.477]***	1.03 [0.926,1.154]
Media exposure	No	2,091 (48.71)	2,201 (51.29)	1.00	1.00
	Yes	1,277 (58.14)	919 (41.86)	1.05 [0.976, 1.133]	1.02 [0.951, 1.091]
Parity	Primiparous	679 (58.48)	483 (41.52)	1.00	1.00
	Multiparous	2,688 (50.48)	2,638 (49.52)	0.68 [0.630, 0.745]***	0.73 [0.6,0.80]***
Family planning	Not used	1,989 (44.29)	2,502 (55.71)	1.00	1.00
	Hormonal	1.285 (69.42)	566 (30.58)	1.92 [1.771,2.082]***	1.63 [1.4, 1.7]***
	Non-hormonal	94 (64.05)	53 (35.95)	1.68 [1.371, 2.071]***	1.37 [1.10,1.69]**
Residence	Rural	2,873 (50.08)	2,864 (49.92)	1.00	1.00
	Urban	494 (65.79)	257 (34.21)	1.69 [1.543, 1.865]***	1.17 [1.03, 1.33]**

AHR, adjusted hazard ratio; CHR, crude hazard ratio; CI, confidence interval, ^**^ = 0.001 ^*^ = p < 0.05, and ^***^ = p < 0.001.

### Spatial and Incremental Autocorrelation Analysis of Resumption of Postpartum Menses Among Women in Ethiopia: Based on 2016 EDHS

**Figure 2:**
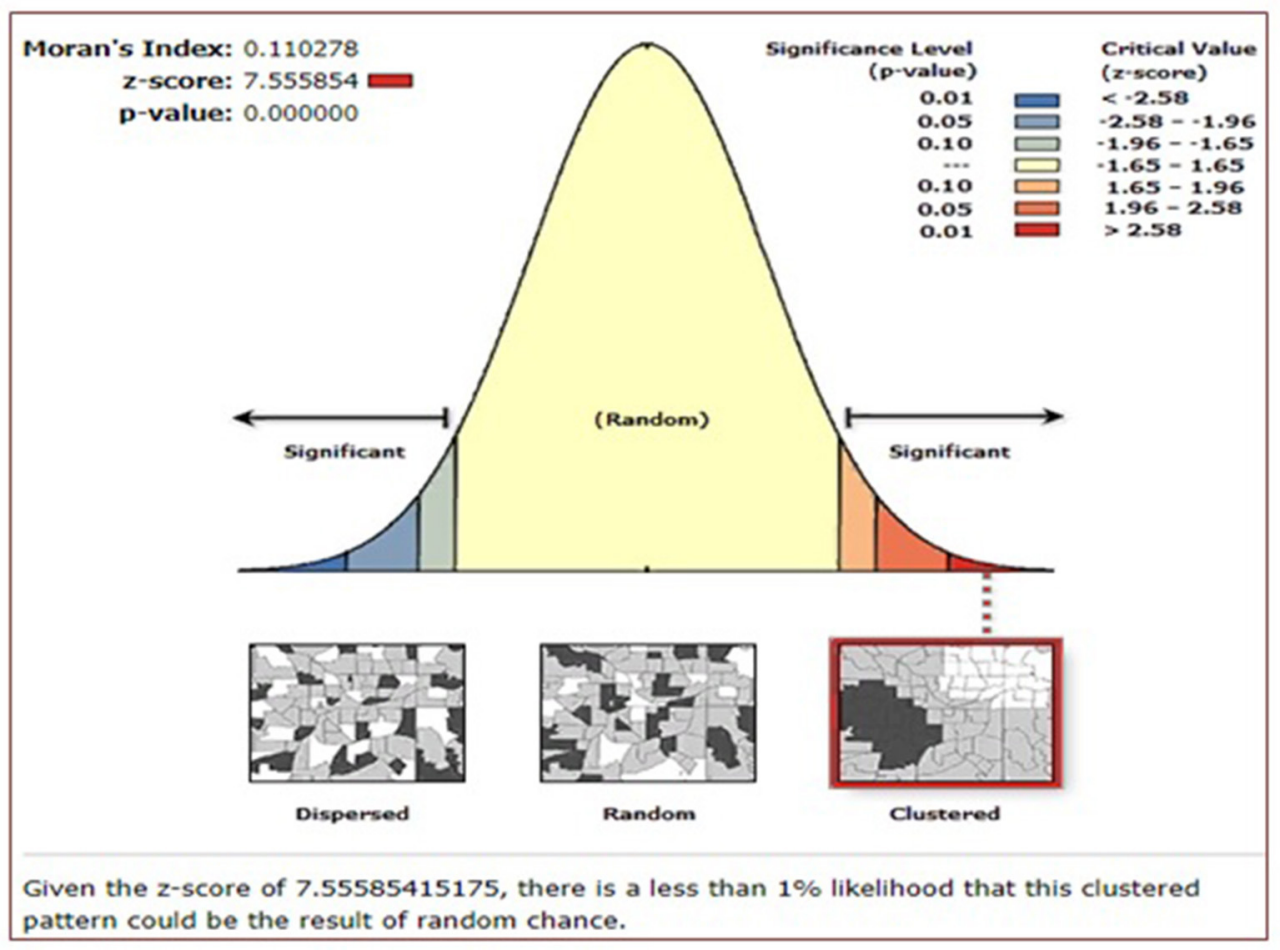
Spatial autocorrelation of resumption of postpartum menses among women in Ethiopia, Ethiopian Demographic and Health Survey (EDHS) 2016.

The spatial distribution of resumption of postpartum menses among women in Ethiopia based on 2016 EDHS showed a significant spatial variation across the country over regions, which was found to be non-random with a Global Moran's *I* value of 0.44 (*p* < 0.0001) (Figure 2).

### Spatial Distribution and Interpolation of Postpartum Menses Resumption in Ethiopia

**Figure 3:**
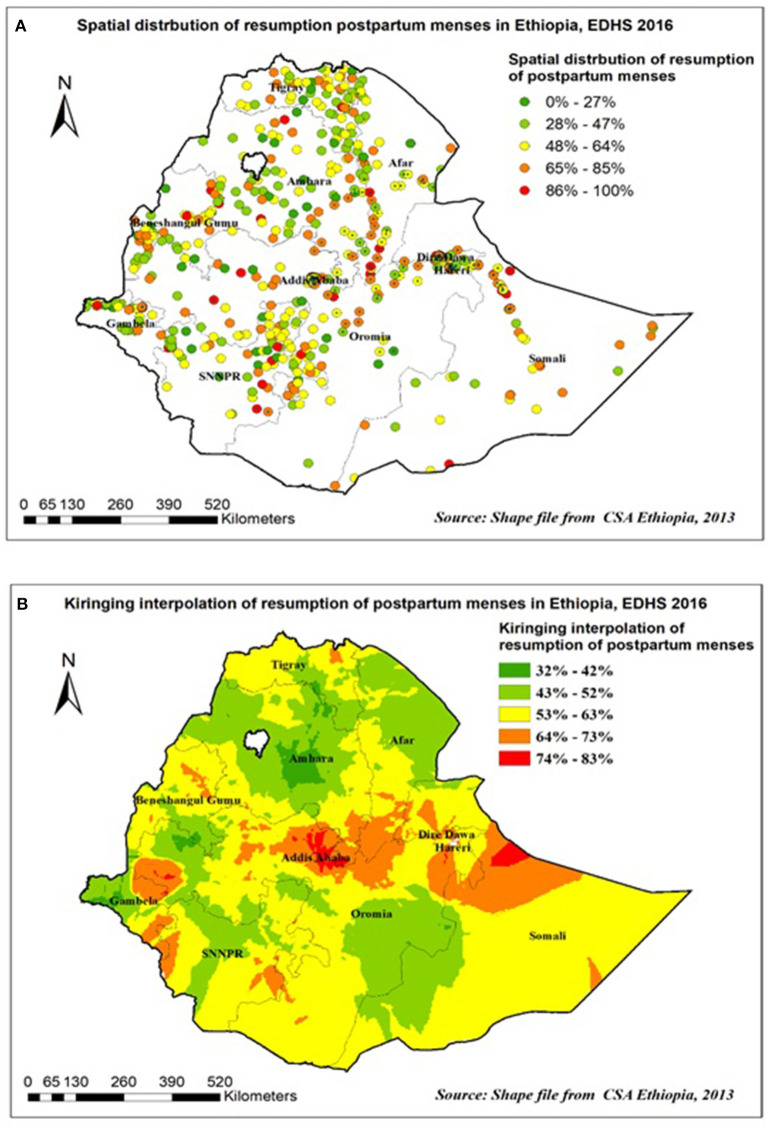
Spatial distribution **(A)** and interpolation **(B)** of resumption of postpartum menses in Ethiopia EDHS 2016.

As shown in the following figures, the red dots indicate the more intense clustering of the proportion of postpartum menses resumption among households in Ethiopia, whereas the green dots show a lower proportion of them (Figure 3A). The prevalence of high-risk areas predicted postpartum menses resumptions was extremely high and ranging from 86 to 100% and was located in Addis Ababa, Dire Dawa, Harare, and the Gambella region. Whereas, Oromia, Amhara, and SNNP (south nation nationalities and peoples of Ethiopia) regions had the lower predicted postpartum menses resumptions (Figure 3B).

Hot spot and spatial SaTScan Statistics analysis of resumption of postpartum menses in Ethiopia.

**Figure 4:**
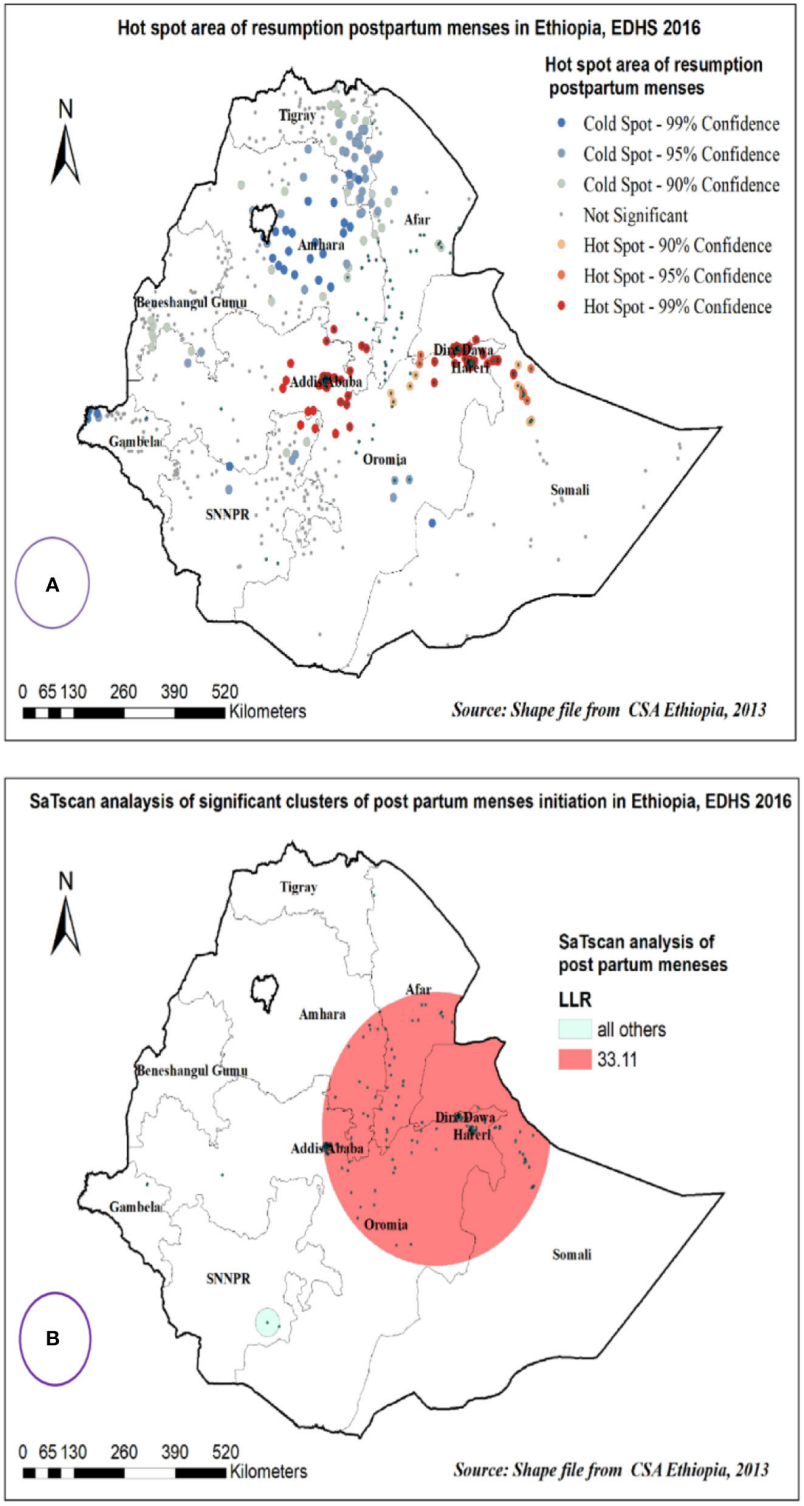
Hot spot area **(A)** and spatial windows **(B)** of postpartum menses resumption in Ethiopia, EDHS 2016.

The spatial distribution of postpartum menses resumption among women in Ethiopia in 2016 EDHS showed that hot spot areas were detected in Addis Ababa and Dire Dawa and Harare, whereas, cold spot areas were detected in Amhara and Tigray region (Figure 4A).

Primary clusters of postpartum menses resumption among women in Ethiopia were identified. Among the total clusters, 231 were primary clusters, which were located in the entire Addis Ababa, Dire Dawa, and Harare regions centered at 9.339604 N, and 41.321415 E with a 292.20 km radius. Women who were found in the SaTScan window were 1.2 times more likely to have postpartum menses (RR = 1.22, *p* < 0.0001) (Table 5, Figure 4B).

**Table 5:** Significant spatial clusters of resumption of post-partum menses among women in Ethiopia, EDHS 2016 data.

**Clusters**	**Enumeration areas (clusters) detected**	**Coordinate/radius**	**Population**	**Cases**	**RR**	**LLR**	**P-value**
1* [231]	3, 491, 333, 372, 441, 557, 453, 594, 25, 412, 30, 43, 380, 644, 546, 282, 74, 190, 111, 151, 273, 631, 101, 140, 467, 535, 363, 519, 471, 5, 613, 166, 606, 390, 185, 444, 506, 385, 224, 27, 202, 493, 514, 352, 311, 473, 476, 523, 242, 281, 607, 173, 443, 115, 610, 614, 383, 393, 179, 28, 60, 228, 133, 56, 29, 397, 238, 500, 329, 157, 257, 418, 58, 396, 580, 240, 513, 387, 534, 44, 495, 587, 381, 483, 194, 321, 288, 357, 419, 68, 642, 454, 501, 212, 436, 622, 564, 39, 307, 336, 1, 51, 566, 135, 230, 49, 122, 37, 71, 245, 186, 529, 484, 8, 210, 102, 57, 64, 439, 283, 295, 624, 620, 276, 201, 637, 277, 568, 547, 319, 527, 121, 22, 310, 55, 123, 116, 75, 125, 452, 239, 334, 251, 573, 570, 596, 149, 40, 440, 33, 368, 214, 632, 286, 290, 213, 524, 303, 254, 499, 178, 572, 205, 472, 345, 18, 90, 402, 521, 287, 553, 588, 458, 211, 560, 562, 509, 330, 428, 155, 611, 423, 247, 19, 464, 639, 353, 15, 153, 83, 264, 293, 170, 582, 539, 451, 112, 414, 144, 236, 61, 252, 305, 302, 159, 635, 532, 617, 225, 463, 4, 369, 108, 110, 626, 475, 195, 261, 31, 59, 107, 100, 11, 339, 274, 191, 314, 645, 487, 608, 145	9.339604 N, 41.321415 E / 292.20 km	**1,971**	1,232	**1.22**	**33.10**	<0.0001

1^*^ = Significant primary clusters.

## Discussion

The timing of the resumption of post-partum menses is indispensable for a woman who intends to avoid subsequent unintended pregnancy and refrain from the complications of abortion and short interbirth interval (28, 40). Acting accordingly at the time of resumption of post-partum menses has key clinical implications on maternal, neonatal, and child health outcomes (40, 41). For those with clinical implications, determining the timing of menses resumption and factors associated with menses return helps inpatient counseling about the ideal time for the initiation of postpartum family planning leading to reduced maternal, neonatal, and child death (42). Moreover, the findings of this study can be used to evaluate the existing clinical guidelines, policies, and strategies for reducing maternal, neonatal, and child deaths due to the complications of the short-interbirth interval and unintended/unwanted pregnancies (43). As a result, this study was aimed to determine the time it takes to start menses, identify its predictors, and explore the spatial distribution among postpartum period women in Ethiopia using 2016 Ethiopian Demographic and Health Survey data.

The overall prevalence of resumption of post-partum menses is 51.90% and the median survival timing of post-partum menses resumption in months is 14.6 in the current study. This finding is in agreement with evidence from National Family and Fertility Survey (NFFS) in Ethiopia where the median time of menses resumption among breastfeeding women was reported as 14 months (42), and the current finding is longer than the other available evidence as menses resumed within a week to 1 month after childbirth for women who do not breastfeed, within 1–2 months for women who breast-and formula feed, and it is normal not to menstruate for 6 months or longer for women who exclusively breastfeed. This was supported by books and the clinical experience of authors (44, 45). The possible explanation for this longer duration for the time of resumption of post-partum menses in the current study compared to the reported evidence by books and clinical experience might be due to the difference in the study population, as the current study is based on aggregate data of an EDHS with a high proportion of post-partum period women with breastfeeding practice. There is evidence that clearly shows rural residency is significantly associated with post-partum breastfeeding practice compared to women residing in the urban areas (46, 47), surprisingly 88.43% of the current study participants are from rural areas. On the other hand, breastfeeding practice is a proximal determinant of post-partum menses resumption, which is supported by shreds of evidence (30, 31, 48). Whereas, evidence from books and clinical experience was based on an individual client-based report and might be highly affected by outliers, reporting the shorter extreme timing of menses resumption might make the shorter finding compared to the current aggregate data.

Regarding the factors associated with the time of resumption of post-partum menses, a total of six variables, including duration of breastfeeding, child alive status, maternal education, parity, hormonal contraceptive use status, and residency, were significantly associated with the outcome variable.

The risk of resumption of post-partum menses among women who have had breastfeeding status for more than 7–24 months (about 2 years) is 36 % less likely compared with those women who never breastfeed. This finding is consistent with evidence reported by various types of literature (49, 50). The possible explanation could be due to the dominance of prolactin hormone among women with a longer duration of breastfeeding and the diminishing of gonadal hormones that are responsible for the surge of ovulation. This hypothesis is supported by evidence as breastfeeding is one of the factors that antagonize the effect of gonadal hormones (estrogen and progesterone) from the proliferation and release of female eggs (51, 52). Therefore, whenever there is no release of eggs or ovulation, there is no menses resumption among post-partum period women.

Post-partum period women with an alive child have a 26% lower risk of menses resumption compared to those who have no child. The current study agrees with the previous study conducted in Indonesia (53), Bangladesh (54), and Senegal (55) that women with stillbirth and early neonatal loss found their post-partum menses had resumed early as compared to women with live children. The possible explanation could be that whenever the child is alive, breastfeeding practice is inevitable, and when there is breastfeeding in the post-partum period, there is inhibition of estrogen and progesterone hormones, which are responsible for the proliferation and release of ovum (56, 57). Therefore, if there is inhibition in the proliferation and release of the ovum, then there is no conception and resumption of menses in the post-partum period. The risks of resumption of post-partum menses among multipara women are 27% less likely to occur as compared to primipara women. This finding is consistent with the evidence from a study done in Mat lab, Bangladesh that showed women with higher parity have a longer duration to resume post-partum menses as compared to women with lower parity (54). The possible explanation could be due to genetic defects in gametes increasing with the increase in age for both men and women (58). There is plenty of evidence about age-related changes in the gonadal–pituitary axis especially, average follicle-stimulating hormone levels appear to increase in the early 30's and become diminished by the late 30's (59, 60). Therefore, mostly multi and grand multipara women are above the age of 35 years, who are highly vulnerable to genetic defects, with less likely to resume menses in the post-partum period.

Women who are using hormonal contraceptives have 1.63 times more risk of resumption of post-partum menses compared to those who did not use hormonal contraceptives. This finding is consistent with the study done in Peru and Indonesia (61). The possible explanation is that the presence of hormonal contraceptives in the bloodstreams of post-partum period women actuates endometrial layer proliferation from the positive feedback of progesterone (62). Therefore, whenever there is no conception, shading of the proliferated endometrial layer is inevitable, and post-partum menses can be resumed. The risks of resumption of post-partum menses among women with formal education are 1.14 times higher compared to women who have no formal education. This finding agrees with the study conducted in Egypt (37), which found that among post-partum women whose menses had resumed, 52% of them were illiterates and 35% of them were secondary and above in terms of education. The possible explanation postulated by scholars is that educated women are less likely to engage in post-partum breastfeeding practice, rather than highly practicing formula feeding for their child (63). Therefore, when the frequency of breastfeeding decreases, follicular hormones, such as estrogen and progesterone, become dominant and post-partum menses can be resumed when there is no conception.

The risk of resumption of post-partum menses is 1.17 times higher among women residing in urban areas compared to those residing in rural areas. The finding of the current study is in line with the previous studies done in Taiwan (64), Zaire (65), and Guatemala (66). The possible explanations could be due to the difference in the culture of breastfeeding practice. Women who reside in the urban area are less likely to engage in breastfeeding practice and it might predispose them to an early resumption of post-partum menses. The other possible explanation is that study participants from the urban areas are modernized in the use of nutritional diversity, and living standards in terms of rest. These might facilitate the return of normal physiological and anatomical structures of the reproductive system and post-partum menses might be resumed early.

## Strength and Limitations of the Study

The main strength of this study was the use of the weighted nationally representative data with a large sample that makes it representative at national and regional levels. Therefore, it can be generalized to all post-partum period women during the study period in Ethiopia. Moreover, the use of a shared frailty model that considered the nested nature of the EDHS data and the variability within the community to get a reliable estimate and standard errors (SEs). However, it is not free of limitations resulting from the use of secondary data as some important confounders, such as the health service quality and behavioral factors, are missed.

## Conclusion

The current study finds that almost half of the study participants had resumed post-partum menses and the median time for the resumption of post-partum menses was found to be longer compared with reports from books, and guidelines. Urban in residency, education, and the use of hormonal contraceptives were variables positively associated with the time of resumption of post-partum menses. On the other hand, breast feeding status, child alive, and multi and grand multiparty were negatively associated with the outcome variable. Therefore, to improve post-partum women's duration of menses resumption, it is better to strengthen strategies focusing on the culture of breastfeeding practice and women's education.

## Data Availability Statement

The original contributions presented in the study are included in the article/supplementary material, further inquiries can be directed to the corresponding author/s.

## Ethics Statement

Ethical review and approval was not required for the study on human participants in accordance with the local legislation and institutional requirements. Written informed consent for participation was not required for this study in accordance with the national legislation and the institutional requirements.

## Author Contributions

DB and MA have done the conception of the work, design of the work, acquisition of data, analysis, interpretation, involved in data curation, drafting the article, revising it critically for intellectual content, validation, and final approval of the version to be published. Both authors contributed to the article and approved the submitted version.

## Conflict of Interest

The authors declare that the research was conducted in the absence of any commercial or financial relationships that could be construed as a potential conflict of interest.

## Publisher's Note

All claims expressed in this article are solely those of the authors and do not necessarily represent those of their affiliated organizations, or those of the publisher, the editors and the reviewers. Any product that may be evaluated in this article, or claim that may be made by its manufacturer, is not guaranteed or endorsed by the publisher.

## Abbreviations

AHR: adjusted hazard ratio
AIC: Akakia Information Criteria
BIC: Bayesian Information Criteria
CI: confidence interval
CSA: Central Statistical Agency
DHS: Demographic and Health Survey
EDHS: Ethiopian Demographic and Health Survey
KR: Kids Record
MOH: Ministry of Health
PNC: Post Natal Care
SDG: Sustainable Development Goal.
